# Procedural pain in routine dental care for children: a part of the Swedish BITA study

**DOI:** 10.1007/s40368-018-0368-2

**Published:** 2018-09-07

**Authors:** M. Ghanei, K. Arnrup, A. Robertson

**Affiliations:** 1 Public Dental Service, Gothenburg, Region Västra Götaland, Sweden; 2 Dental Research Department, Public Dental Service, Örebro, Region Örebro County, Sweden; 3 School of Health Sciences, Örebro University, Örebro, Sweden; 4 Department of Pediatric Dentistry, Institute of Odontology at the Sahlgrenska Academy, University of Gothenburg, P.O. Box 450, 405 30 Gothenburg, Sweden

**Keywords:** Pain, Dental, Children, Discomfort

## Abstract

**Aim:**

To investigate the frequency and reported intensity levels of dental treatment pain and discomfort in children, in conjunction with regular dental visits.

**Methods:**

The study included 2363 children in four different age cohorts. Data was collected from structured interviews by dental personnel regarding pain experiences or discomfort after treatments, including analgesia, extractions, operative treatments and radiographic examinations.

**Results:**

One-third of all treatment occasions were experienced as painful and/or causing discomfort. Treatment sessions including analgesia were assessed as painful in 49.7% of occasions, with injection being the most common given reason for pain. Extraction was painful in 62.4% of occasions, with injection as the main reason for pain. Operative treatments were assessed as painful in 38.8% of occasions, with drilling as the most common reason for pain and discomfort. Pain was reported in approximately 19% of all radiographic examinations.

**Conclusions:**

Injection was the major reason for pain during treatment, including injection and extraction, while drilling was the most common cause of pain during restorative treatment. Dentists should try to minimise the experience of pain and discomfort by using all available measures to perform pain-free and effective dental injections.

## Introduction

Pain is defined as an unpleasant sensory or emotional experience arising from actual or potential tissue damage (Loeser and Treede 2008). Patients have commonly described dental procedures as painful and unpleasant. Pain control is important and necessary for successful paediatric dental care.

It is important for dentists to listen to and be aware of their patients’ responses so that treatment can be managed as pain-free as possible. Murtomaa et al. (1996) found that dentists routinely did not question children about pain, but encouraging the child to report pain during treatment. Versloot et al. (2004) studied pain assessed by the child itself, and by dentists and independent observers, during dental injection in children aged 4–8 years, treated at a special dental care centre in Amsterdam, and at a private dental practice specialising in treating children. Dentists assessed the pain significantly lower than the children and observers did. The dentists believed the child to be distressed rather than experiencing pain at that moment. A questionnaire study by Wondimu and Dahllof (2005) regarding attitudes to pain among general dental practitioners in Sweden, concluded that dentists underuse local anaesthetics, analgesics, and sedatives for pain management, during the dental treatment of children and adolescents. In another questionnaire study in Denmark in 2005, regarding knowledge, attitudes and management of pain during paediatric dental care, one-quarter of the responding dentists working with children in a municipal dental service or in private practice, answered that children could report pain only with uncertainty. A large proportion agreed to the statement that “learning to cope with slight pain is part of life” and that “complete painlessness is a utopia” (Rasmussen et al. 2005).

Nakai et al. (2000) found that ineffective pain control was relatively common, even in specialised paediatric practices, thus strengthening the importance of reducing the patients’ anxiety and peri-operative infections and symptoms to improve pain control. Despite this, the attitudes among dentists remain questionable and the underuse of pain control seems to continue (Wondimu and Dahllof 2005; Ronneberg et al. 2015).

Although the use of local analgesics prevent further procedural pain, the delivery is known to be uncomfortable and may provoke anxiety, particularly in children (Ram and Peretz 2002). In fact, dental injection has been identified as one of the most painful components in dental treatment (Nakai et al. 2005; Krekmanova et al. 2009; Naoumova et al. 2012). For example, the experience of injection was graded worse than the extraction in a study of pain, discomfort and dental anxiety following the extraction of primary canines in 44 children, aged 10–13 years, with palatally displaced canines. There was also a strong positive relationship between dental anxiety scale and pain during injection and extraction (Naoumova et al. 2012). Different parts of the mouth vary in the discomfort reported after injection. Some studies show that mandibular injections are more uncomfortable than those in the maxilla, although other studies report contradictory results (Jones et al. 1995; Ram and Peretz 2001; Versloot et al. 2008; Aminabadi et al. 2009; Meechan 2009).

Discomfort during radiographic examinations has been reported in some studies (Pitts et al. 1991; Pierro et al. 2008; Novaes et al. 2012). Both the use of a holder or the film alone has been reported to cause some discomfort in children (Pitts et al. 1991; Pierro et al. 2008).

There are different methods for children to report their pain experience. The usefulness of different scales to assess pain in children has been extensively studied (Hain 1997). For children between 4 and 6 years of age, an adapted self-report (facial scales), combined with some form of behavioural rating is the most common method (Haukali et al. 2017). For children from about the age of seven, the visual analogue scale (VAS) has been found reliable and recommended for use (McGrath et al. 1996; Young 2005).

Although the most pain-provoking treatment steps are known, little work has been published to quantify the intra-operative pain intensity of routine treatment in general dental practice, with children and adolescents. Thus, the aim of this study was to investigate the frequency and reported intensity levels of dental treatment pain and discomfort in children and adolescents, 3–19 years of age, receiving routine dental care.

## Materials and methods

### Project description

The BITA project (BITA-study (Barn I TAndvården = Children in dental care)) is a 5-year cohort study (accelerated longitudinal design) of children and adolescents (3–19 years old) in the Swedish Public Dental Service. Data collection began in 2008 and ended in 2012. The BITA study concerns different aspects of children in the dental situation when attending regular general dentistry. An overall aim was to achieve a deeper understanding of dental fear and dental behaviour management problems in a context of general dentistry, in Sweden.

### Patients

This study is based on a representative sample of children and adolescents aged 3–19 years in the Region Västra Götaland (RVG) and Region Örebro County (ROC). Four age cohorts with children aged 3, 7, 11 and 15 years in 2008, from seven Public Dental Service clinics in RVG and from five in ROC, were invited to participate and then followed over a 5-year period in conjunction with regular dental visits. The clinics were selected to reflect the respective populations and to cover urban and rural areas.

A total of 3134 children were considered for the study. Of these, 771 declined to participate, whereby 2363 children, in the four age cohorts, became eligible for the study. These four age cohorts included Cohort 1 (born 2005; from 3 to 7 years old during the study period; n = 695 children, 3906 visits), Cohort 2 (born 2001; 7–11 years old; n = 642 children, 4588 visits), Cohort 3 (born 1997; 11–15 years old; n = 574 children, 4085 visits), and Cohort 4 (born 1993; 15–19 years old; n = 452 children, 3315 visits). The distribution between the genders was 1215 girls (51%) and 1148 boys (49%).

### Procedure

#### Clinical registrations

The children’s ordinary dentists registered all performed dental treatment after each session. For this study, four common treatment type sessions were selected; (1) treatments with injection, (2) treatments with extraction, (3) operative treatments including restorative treatments, enamel preparation and fissure sealant, approximal grinding and orthodontic grinding therapy, and (4) radiographic examinations (Table 1).

**Table 1:** Number and percentage distribution of patients (total n = 2363) undergoing the four studied treatment type sessions at least once during the study period 2008–2012

Procedure	Patients count (%)	Sessions count (%)
Treatment with injection	592 (25.1)	1058 (6.7)
Treatment with extraction	308 (13.0)	436 (2.7)
Operative treatment	643 (27.2)	1202 (7.6)
Radiographic examination	1794 (75.9)	4860 (30.1)

Number of each treatment type session and percentage proportion of the total number of sessions (n = 15,894)

#### Assessments of pain and discomfort

Data was collected from structured interviews by dental personnel regarding pain experiences or discomfort (frequency and intensity), after each treatment session. The child was first asked the question, “*Was something painful*?” and/or “*Did you feel any discomfort*?” Yes or no? If yes, children above the age of seven were asked to grade the pain intensity on a visual analog scale (VAS). The scale resembles a thermometer and consists of 11 points running from 0 = no pain/discomfort, to 10 = worst pain/discomfort possible. For children under the age of seven years, the scale contained six faces pictured to express different levels of pain/distress. Finally, the child was asked the question, “*What caused the pain?*” *and*/*or* “*What caused the discomfort?*”

### Ethics

An application for ethical review (No. 286-07) was submitted in June 2007; however, according to the advisory opinion, the Ethics Review Committee found the project not subject to the Swedish Act on Ethical Review. The Committee primarily commented on the information given to patients, which was taken into account during further planning of the study. The subjects also gave their written consent to participate in the research project.

### Statistical methods

In addition to descriptive statistics in terms of frequency distributions, means, and standard deviations, differences between genders and between cohorts were analysed using Chi square tests. The statistical analyses were performed in IBM SPSS Statistics for Windows, Version 23.0 (IBM Corp., Armonk, NY, USA) and *p* values below 0.05 represented statistical significance.

## Results

In total, there were 2363 patients comprising 15,894 treatment occasions (Table 1). For the four treatment-type sessions, pain and discomfort assessments were completed for 99 and 95%, respectively. In 19% of the treatment occasions, something was assessed as painful and discomfort was reported in 18% of the treatment occasions. In 33% of the treatment occasions, something was assessed as painful and/or uncomfortable.

### Pain and discomfort in treatments including injection of local analgesia

Half of the treatment sessions where analgesia was included were assessed as painful (49.7%; Table 2), with the most common given reason for pain being the injection (351/522, 67.2%). Discomfort was reported in 32.4% of the sessions including analgesia, with the injection itself being the reason given in 44%; other aspects such as the use of topical analgesia, numbness caused by the injection, analgesics drained into throat, and the residual numbness after injection, emerged as reasons for discomfort in 66% of those sessions.

**Table 2:** Reports of pain and/or discomfort during treatment sessions including injection (n = 1058), extraction occasions (n = 436), operative treatments (n = 1202) and radiographic examinations (n = 4860)

	Valid n	Pain (%)	*p* value	Valid n	Discomfort (%)	*p* value
Injection						
Total	1051	49.7		1014	32.4	
Girls	582	51.7	0.138	557	35.4	*0.028*
Boys	469	47.1		457	28.9	
Cohort 1	128	46.1	*0.001*	125	26.4	0.109
Cohort 2	368	53.3		354	33.1	
Cohort 3	308	55.2		298	36.9	
Cohort 4	247	39.3		237	29.1	
Extraction						
Total		433	62.4		420	33.3
Girls	231	63.2	0.697	224	37.1	0.084
Boys	202	61.4		196	29.1	
Cohort 1	39	59.0	0.435	37	29.7	*0.001*
Cohort 2	167	66.5		164	33.5	
Cohort 3	181	61.3		173	38.7	
Cohort 4	46	54.3		46	15.2	
Operative treatment						
Total	1198	38.8		1155	28.5	
Girls	654	40.7	0.148	628	30.6	0.086
Boys	544	36.6		527	26.0	
Cohort 1	167	33.5	*0.005*	164	20.7	0.073
Cohort 2	396	44.9		382	29.1	
Cohort 3	311	39.9		301	32.2	
Cohort 4	324	33.0		308	28.2	
Radiographic examination						
Total	4823	19.3		4688	17.7	
Girls	2464	21.1	*0.001*	2393	18.1	0.576
Boys	2359	17.4		2295	17.4	
Cohort 1	643	12.9	*0.000*	638	16.1	*0.002*
Cohort 2	1557	26.7		1525	19.1	
Cohort 3	1381	19.0		1330	19.8	
Cohort 4	1242	13.8		1195	14.6	

Differences between genders and between cohorts were tested using Chi square tests and significant *p*-values are given in italics

There were no significant differences between genders regarding the reporting of pain in treatments where injection was a part, but discomfort was more often reported among girls than boys (35.4% vs 28.9%; *p* = 0.028; Table 2).

In reporting pain during treatments including analgesia, there was a statistically significant difference between cohorts, with the lowest proportions of pain reports from the youngest and oldest groups (46%, 53%, 55%, 39%; *p* = 0.001), while cohort differences in discomfort reports were non-significant (Table 2).

In the treatment sessions including injection (children with no pain excluded), the mean intensity of pain was 3.75 (SD 2.210). In the same way, the mean intensity of discomfort was 3.55 (SD 2.191; Table 3).

**Table 3:** Assessment of pain intensity in patients who indicated something was painful or creating discomfort during at least one occasion of injection, extraction, operative treatment or radiographic examinations

Treatment type sessions and moments	Valid n (sessions)	Mean	SD	Range
Treatments with analgesia				
Injection painful	330	3.75	2.210	0–10
Injection uncomfortable	134	3.55	2.191	0–10
Tooth extractions				
Extraction painful	41	4.79	2.437	1–10
Injection painful	184	3.56	1.987	0–10
Extraction and injections painful	15	5.67	2.177	2–10
Extraction uncomfortable	56	3.58	1.969	0–8
Injection uncomfortable	42	3.76	2.385	1–9
Extraction and injection uncomfortable	4	5.63	4.110	1–10
Operative treatment				
Drilling painful	213	3.74	2.235	0–10
Injection painful	169	3.52	2.183	0–10
Drilling and injection painful	10	4.55	2.544	2–10
Drilling uncomfortable	103	3.31	1.988	0–10
Injection uncomfortable	101	3.40	1.994	0–10
Drilling and injection uncomfortable	5	4.00	2 646	1–6
Radiographic examination				
Radiographic examination painful	556	3.58	2.021	0–10
Radiographic examination uncomfortable	380	3.35	2.041	0–10

### Pain and discomfort in treatments with extraction

Of all treatments including extraction, 62.4% were assessed as painful (Table 2). The reasons given for pain were the extraction (15.9%), the injection (71.5%) or the combination of injection and extraction (6.3%).

Discomfort was reported in 33.3% of the occasions where extraction was a part. The reasons reported for discomfort during extraction pointed more clearly to the extraction per se (42.1%), followed by injection (33.6%) or a combination (3.6%).

No statistically significant difference between the genders or cohorts was found regarding the experience of pain. In reporting discomfort during extractions, there was a statistically significant difference between cohorts, with the lowest proportion in the oldest age group (Table 2*)*.

Primary and permanent tooth extractions were assessed as painful in equal proportions (62.8% and 61.1%), while discomfort was slightly less often reported for primary than for permanent tooth extractions (32.4% and 37.1%).

The mean intensity of pain in children who indicated extraction as the reason was 4.79 (SD 2.437), the mean intensity of pain in children who indicated injection as the reason was 3.56 (SD 1.987), and the mean intensity of pain in children who stated both injection and extraction as the reason for pain was 5.67 (SD 2.177) (Table 3). The mean intensity of discomfort ranged from 3.58 to 5.63 (Table 3).

### Pain and discomfort during operative treatments

Operative treatments included restorative treatment (1078 treatments), enamel preparation and fissure sealant (30 treatments), enamel preparation, fissure sealant and restoration (9 treatments), approximal grinding (11 treatments), and orthodontic grinding therapy (59 treatments). Eleven treatment sessions were unspecified. The most performed treatment in this group was an enamel-dentine preparation and a filling (n = 1078, 90%).

Pain was reported in 38.8% of these treatment occasions with drilling being the most common given reason for pain (47.1%), followed by injection (38.5%), or drilling and injection combined (2.2%). Discomfort was reported in 28.5% of the operative treatment occasions with drilling (34.0%), injection (31.6%), or both (1.8%) as reasons.

Pain reports differed significantly between cohorts (Table 2), following the same pattern as for pain in treatment sessions including injection, while differences regarding discomfort were non-significant (Table 2). The difference between genders during operative treatment was not statistically significant.

In separating operative treatments with (58.4%) or without (41.6%) local analgesia, the proportions of sessions assessed as painful were 43.6% and 32.1%, respectively. Furthermore, considering the whole spectra of painful or not painful treatments, with or without local analgesia (Table 4), it was revealed that 13.4% of all operative treatments were painful treatments without analgesia, and 25.5% of the treatments were painful although local analgesia had been given.

**Table 4:** Experience of pain related to operative treatment performed with or without analgesia. Percentages are given as proportions of all operative treatments (valid n = 1198)

	Not painful	Painful	Total
With analgesia	395 (32.9%)	305 (25.5%)	700 (58.4%)
Without analgesia	338 (28.2%)	160 (13.4%)	498 (41.6%)
All	733 (61.2%)	465 (38.8%)	1198

During operative treatments, the mean intensity of pain was 3.74 (SD 2235) in patients who indicated drilling was painful, 3.52 (SD 2183) in patients who indicated that injection was painful, and 4.55 (SD 2.544) in patients who stated both injection and drilling were painful (Table 3). The mean intensity of discomfort ranged from 3.41 to 3.10 (Table 3).

### Pain and discomfort during radiographic examinations

Pain or discomfort in conjunction with radiographic examinations was reported in 19.3 and 17.7% of the sessions, respectively (Table 2). The radiographic procedure itself was given as the reason for pain in 65.2% and for discomfort in 49.7%. The most frequent reporting of pain related to radiographic examination was given by the cohort aged seven years at study start, while discomfort differences between cohorts, although statistically significant, were less pronounced (Table 2). Girls reported pain significantly more often than boys (Table 2). Discomfort was reported in equal proportions by girls and boys.

In patients who indicated that radiographic examinations were painful, the mean pain intensity was 3.58 (SD 2,021). In the same way, the mean intensity of discomfort was 3.35 (SD 2.041) (Table 3).

## Discussion

This study has shown that approximately one-third of all treatment occasions were reported as painful and/or causing discomfort. When performed, injection emerged as the main cause for pain, also when extraction followed. A second common reason was drilling. The present study is the first to examine the reporting of pain and discomfort over a five-year period, within regular dental care, for a large number of patients (3–19 years old).

The injection, having emerged as the most common reason for pain, also when other treatments using analgesia were performed, is in agreement with other studies pointing to the dental injection as the highest ranked painful dental treatment event (Nakai et al. 2005; Krekmanova et al. 2009; Naoumova et al. 2012).

Many children reported injection as painful during extraction. This is in agreement with Naoumova et al. (2012), who reported that the experience of injection was graded worse than the primary canine extraction itself. However, the mean intensity of pain, reported in this BITA sample, was higher when extraction was given as the reason for pain, and even higher when the reason was extraction and injection combined, as compared to when the reason was injection alone. This could be a sign of insufficient administration of analgesia during this procedure.

The present study showed that approximately one-third of all operative treatment occasions were performed with analgesia and were not painful, which is the optimal wish for all children receiving restorative treatment; although, more than one-quarter of all operative treatment occasions were both performed with analgesia and were reported as painful by the children. In addition to painful injection, reasons that may explain pain during restorative treatment could be insufficient analgesia, or if drilling is performed directly after analgesia, without waiting to achieve an adequate anaesthetic effect.

Children reported painful treatment in nearly one-third of the operative treatment occasions which were performed without analgesia. It is important to motivate these patients to take analgesia. There are also dentists who do not believe that analgesia is necessary for all restorative treatments. A study by Rasmussen et al. (2005), using a questionnaire, showed that 57.5% of the dentists agreed to perform filling of a superficial cavity in 36 without local analgesia. A recent study showed that dentists reported a less frequent use of local analgesia in children younger than 10 years of age, when performing restorative treatment (Ronneberg et al. 2015).

The overall frequency and mean intensity of discomfort was lower than the mean intensity of pain during dental procedures. However, there is a group of children who reported only discomfort, or a combination of pain and discomfort, during dental treatments. Thus, it is important to not only reduce the experience of pain, but also minimise the perception of discomfort during dental treatments.

Radiographic examinations were performed in volume and the reporting of pain and discomfort was less frequent than for invasive treatment procedures. However, for those who reported pain related to radiographic examinations, the mean pain intensity was almost the same as for injections. Other studies have examined only the experience of discomfort during radiographic examinations, since radiographic examinations are mostly linked to being more uncomfortable than causing pain (Pitts et al. 1991; Pierro et al. 2008; Novaes et al. 2012). Thus, although the results were in concordance with earlier studies reporting a high acceptance rate and a low degree of discomfort with radiographic examinations (Pitts et al. 1991; Novaes et al. 2012), the risk for pain from radiographic examinations should not be overlooked. Radiographic examinations involved many children during several occasions. The results point to the importance of performing this procedure as painless as possible.

There were no significant gender differences regarding the experience of pain during invasive dental treatments in this study, which is in line with other studies (Jones et al. 1995; Ram and Peretz 2001; Allen et al. 2002, 2003; Aminabadi et al. 2009; Naoumova et al. 2012), however, some studies have shown that girls experience dental injection significantly more painful than boys (Peretz and Efrat 2000; Nakai et al. 2005; Krekmanova et al. 2009).

The child’s age had an association with the report of pain and discomfort in the present study, with the highest frequencies reported for ages 7–15 years. An explanation could be that the experience of invasive treatment is often limited for the youngest children. Fewer treatments are performed at this age due to having good dental health in Sweden. For many children, the first experiences of invasive treatments are usually after 7 years of age, e.g., in this study, most tooth extractions were performed on children aged 7–15 years.

Children, aged 7–11 years, reported significantly more pain than other children during radiographic examinations. Often, standard-size radiographic films or plates are used and for younger children, with the mouth still small in relation to the size of the film/plate. This, together with the sharp edges of the film or film holder, may cause pain and discomfort in children. A higher bitewing acceptance rate for older than for younger children (10–15 years vs. 5–9 years), has also been reported by Pitts et al. (1991).

Treatments were performed by many dentists from several Public Dental Service clinics during a 5-year period, which is the strength of this study, since it shows it is realistic to everyday dentistry, even though the information may not be completely accurate at all times. One limitation of this study was that radiographic examinations were performed with digital receptors in only some of the dental clinics. No comparison in children’s reporting of pain and discomfort between conventional film and digital receptors was made. This study has assessed each estimate separately. Some children may have experienced pain in connection to one treatment. No comparison has been made to see the influence of negative dental experience during subsequent treatment sessions. Versloot et al. studied 147 children (4–11 years of age) and explored the influence of age, previous dental experiences, level of dental anxiety, and the injection site on self-reported pain during the first and second treatment sessions. For both younger and older children, higher pain scores the first time predicted higher scores the second time (Versloot et al. 2008).

The fact that children aged 7–15 years had rated their experience of dental treatment as painful shows the necessity to have even more effective pain control in this age group, in order to reduce the negative pain experience and the child’s anxiety in the future. For many children, extractions due to orthodontic treatment or other causes can be their first experience of invasive dental treatment. For that reason, it is important to introduce and prepare children before starting treatment and performing painless analgesia. There are a number of things that a dentist can use to reduce the expectation of pain and lessen the child’s anxiety during the administration of local analgesia; the use of topical analgesics prior to injection, slow injection technique, the use of transpapillary injection and behaviour management methods.

## Conclusions

This study has shown that one-third of all treatment occasions were reported as painful and/or causing discomfort. Injection was, when included, still the main reason for pain. Injection was the dominant reason for pain during extraction, while drilling was the most common cause of pain during restorative treatment. Thus, dentists should try to minimise the experience of pain and discomfort by taking all means to introduce and perform pain-free and effective dental injections.

## References

[CR1] Allen KD, Kotil D, Larzelere RE, Hutfless S, Beiraghi S (2002). Comparison of a computerized anaesthesia device with a traditional syringe in preschool children. Pediatr Dent.

[CR2] Aminabadi NA, Farahani RM, Oskouei SG (2009). Site-specificity of pain sensitivity to intraoral anesthetic injections in children. J Oral Sci.

[CR3] Hain RD (1997). Pain scales in children: a review. Palliat Med.

[CR4] Haukali G, Lundeberg S, Hogsbro Ostergaard BDH, Pain, Koch G, Poulsen S, Espelid I, Haubeck D (2017). Pain control. Paediactric dentistry—a clinical approach.

[CR5] Jones CM, Heidmann J, Gerrish AC (1995). Children’s ratings of dental injection and treatment pain, and the influence of the time taken to administer the injection. Int J Paediatr Dent.

[CR6] Krekmanova L, Bergius M, Robertson A (2009). Everyday- and dental-pain experiences in healthy Swedish 8–19 year olds: an epidemiological study. Int J Paediatr Dent.

[CR7] Loeser JD, Treede RD (2008). The Kyoto protocol of IASP basic pain terminology. Pain.

[CR8] McGrath PA, Seifert CE, Speechley KN (1996). A new analogue scale for assessing children’s pain: an initial validation study. Pain.

[CR9] Meechan JG (2009). Pain control in local analgesia. Eur Arch Paediatr Dent.

[CR10] Murtomaa H, Milgrom P, Weinstein P, Vuopio T (1996). Dentists’ perceptions and management of pain experienced by children during treatment: a survey of groups of dentists in the USA and Finland. Int J Paediatr Dent.

[CR11] Nakai Y, Milgrom P, Mancl L (2000). Effectiveness of local anaesthesia in paediatric dental practice. J Am Dent Assoc.

[CR12] Nakai Y, Hirakawa T, Milgrom P (2005). The Children’s Fear Survey Schedule-Dental Subscale in Japan. Community Dent Oral Epidemiol.

[CR13] Naoumova J, Kjellberg H, Kurol J, Mohlin B (2012). Pain, discomfort, and use of analgesics following the extraction of primary canines in children with palatally displaced canines. Int J Paediatr Dent.

[CR14] Novaes TF, Matos R, Raggio DP, Braga MM, Mendes FM (2012). Children’s discomfort in assessments using different methods for approximal caries detection. Braz Oral Res.

[CR15] Peretz B, Efrat J (2000). Dental anxiety among young adolescent patients in Israel. Int J Paediatr Dent.

[CR16] Pierro VS, Barcelos R, de Souza IP, Raymundo RJ (2008). Paediatric bitewing film holder: preschoolers’ acceptance and radiographs’ diagnostic quality. Pediatr Dent.

[CR17] Pitts NB, Hamood SS, Longbottom C, Rimmer PA (1991). The use of bitewing positioning devices in children’s dentistry. Dentomaxillofac Radiol.

[CR18] Ram D, Peretz B (2001). Reactions of children to maxillary infiltration and mandibular block injections. Pediatr Dent.

[CR19] Ram D, Peretz B (2002). Administering local anaesthesia to paediatric dental patients—current status and prospects for the future. Int J Paediatr Dent.

[CR20] Ram D, Peretz B (2003). The assessment of pain sensation during local anaesthesia using a computerized local anaesthesia (Wand) and a conventional syringe. J Dent Child (Chic).

[CR21] Rasmussen JK, Frederiksen JA, Hallonsten AL, Poulsen S (2005). Danish dentists’ knowledge, attitudes and management of procedural dental pain in children: association with demographic characteristics, structural factors, perceived stress during the administration of local analgesia and their tolerance towards pain. Int J Paediatr Dent.

[CR22] Ronneberg A, Strom K, Skaare AB, Willumsen T, Espelid I (2015). Dentists’ self-perceived stress and difficulties when performing restorative treatment in children. Eur Arch Paediatr Dent.

[CR23] Versloot J, Veerkamp JS, Hoogstraten J (2004). Assessment of pain by the child, dentist, and independent observers. Pediatr Dent.

[CR24] Versloot J, Veerkamp JS, Hoogstraten J (2008). Children’s self-reported pain at the dentist. Pain.

[CR25] Wondimu B, Dahllof G (2005). Attitudes of Swedish dentists to pain and pain management during dental treatment of children and adolescents. Eur J Paediatr Dent.

[CR26] Young KD (2005). Paediatric procedural pain. Ann Emerg Med.

